# Trajectories of growth and language development among children with congenital hypothyroidism concerning gender differences: Egyptian study

**DOI:** 10.1186/s12887-026-06635-7

**Published:** 2026-04-02

**Authors:** Hassnaa Othman Mohammed, Lamis Hassan Emad Mekkawy, Rehab Khaleel Ali, Omar El Sayed El-Shourbagy

**Affiliations:** 1 Medical Studies Department for Children, Faculty of Postgraduate Childhood Studies, Ain Shams University, 45-B Souzan Moubarak street el-Qobba, Cairo, Egypt; 2 Pediatrics and Endocrinology Consultant at VACSERA Diabetes Center, Cairo, Egypt

**Keywords:** Congenital hypothyroidism, Developmental assessment, Growth chart, Language assessment, Cognitive assessment

## Abstract

**Background:**

Despite significant advancements in the early detection and treatment of congenital hypothyroidism (CH), a developmental and growth gap persists between affected children and their healthy peers. This study aims to assess some growth and developmental aspects among a sample of Egyptian children who had CH and were subjected to the recommended national CH treatment program, with a focus on age- and gender-related differences.

**Methods:**

A prospective case-control follow-up study was conducted from February 2021 to February 2023 in Giza Governorate, Egypt, involving 80 children aged 1.5–3 years, including 40 with CH (Group A) and 40 age- and gender-matched healthy controls (Group B). CH was diagnosed at birth via newborn screening and confirmed by elevated TSH and low free T4 levels. Participants underwent two follow-up assessments: the first one year after initiation of thyroid hormone therapy and confirmation of treatment adherence, and the second one year later. During each follow-up, clinical, laboratory, growth, and developmental evaluations were conducted using CDC growth charts, PLS-4 (Arabic Version), Stanford-Binet 5th Edition, and Portage Program Questionnaire.

**Results:**

By the second follow-up, children with CH showed significant delays across all developmental domains compared to controls (*p* ≤ 0.001). Female children with CH exhibited earlier language delays—both receptive (*p* = 0.025) and expressive (*p* = 0.015)—during the first follow-up. In the second follow-up, girls continued to show deficits in height, weight, and receptive language age, although expressive language showed signs of improvement. Male children demonstrated more pronounced growth and developmental compromise during the second follow-up, with significant deficits in height (*p* = 0.001), weight (*p* = 0.016), and receptive language age (*p* = 0.001). An inverse correlation was observed between receptive language age and CH status.

**Conclusions:**

Children with CH continue to face developmental and growth challenges despite adherence to standard treatment protocols. Delays appear earlier in girls, particularly in language domains, whereas boys tend to show more pronounced deficits with increasing age. These findings indicate a distinct gender-based developmental pattern in children with CH, emphasizing the importance of sex-specific monitoring and timely interventions to optimize long-term outcomes.

## Background

Congenital hypothyroidism (CH) is a pediatric endocrine disorder characterized by insufficient thyroid hormone production, typically resulting from thyroid dysgenesis or dyshormonogenesis. If diagnosed and treated early, CH is one of the leading causes of preventable intellectual disability [1, 2]. The Egyptian Ministry of Health and Population has implemented a neonatal screening program, achieving a coverage rate exceeding 90% for the early detection of CH [3, 4]. The incidence of CH is approximately 1 in every 3,000 to 4,000 newborns, with a prevalence that is two to three times higher in females. In 2005, Egypt reported a higher incidence of 1 in 2,020 live births [5, 6]. In recent years, there has been a global increase in CH incidence [7], which can be attributed to several factors, including the reduction in screening cutoff levels for thyroid-stimulating hormone (TSH), improved survival rates following neonatal intensive care unit (NICU) admissions, and environmental factors such as iodine deficiency [8, 9]. The etiology of CH remains largely unknown. Hakim [10] emphasized the importance of continuously investigating modifiable risk factors to develop effective prevention and early intervention strategies. Abdelmoktader [11] identified several risk factors for CH in El-Fayoum, including congenital disabilities, female gender, gestational age over 40 weeks, and gestational diabetes. The course of CH can be either permanent or transient. Transient Congenital Hypothyroidism (TCH) typically resolves spontaneously within a few months or years of life. Currently, there is a lack of reliable markers to predict TCH at the time of diagnosis, and the condition is confirmed after the withdrawal of levothyroxine therapy around 3 years of age [12, 13]. The incidence of TCH is increasing and is a significant factor contributing to the overall rise in the incidence of CH in recent studies [14]. Predisposing factors for TCH include preterm birth, low birth weight, male sex, and admission to NICUs [15, 16]. Males are more commonly affected by the transient form, whereas the permanent form is more prevalent in females [17].

Thyroxine (T4) and TSH play crucial roles in musculoskeletal development, brain growth, and overall body homeostasis. Their actions involve central nervous system processes such as vascularization, myelination, dendritic arborization, cell differentiation, and gene expression [18, 19]. Hormone deficiency in brain regions responsible for cognitive functions can result in delays in oral language production, comprehension difficulties, and reduced vocabulary, as well as morpho-syntactic changes [20, 21], which eventually can significantly hinder the social integration of children with CH [22, 23].

Razón-Hernández et al. [24] reviewed the literature and concluded that CH is a chronic condition with long-lasting effects. In neonates and infants, CH is associated with deficits in sensorimotor skills, particularly impaired balance. In preschool and elementary school children, as well as adolescents, CH leads to alterations in intellectual quotient (low scores), language acquisition, memory (including visual, verbal, visuospatial, and semantic memory), and sensorimotor skills (with impaired fine and gross motor control). Additionally, visuoconstructive and visuospatial abilities are affected, as evidenced by low scores in tasks like spatial location, block design, and object assembly. These neuropsychological impairments extend into young adulthood, with the exception of language skills, where verbal fluency remains relatively intact. Despite early hormone replacement therapy being recognized as a protective factor against intellectual disability, gaps persist in understanding the full impact of CH on child development [25]. Gejão et al. [26] recommended a multidisciplinary team to monitor and manage children with CH. This team typically includes a pediatrician, endocrinologist, nutritionist, psychologist, neurologist, social assistant, phoniatrician, audiologist, and biochemist to provide comprehensive care for children.

The increased incidence rate of CH among children, especially in our community, together with unsatisfactory growth and developmental gain among children with CH despite the best regimen of management of the disorder, highlighted the importance of continuous and multidisciplinary assessment of these children in order to promote the growth and neurocognitive outcome among this population.

The objective of the current study was to conduct a detailed, objective, and repeated follow-up on various growth and developmental domains in Egyptian children with CH, who are receiving the conventional management plan.

## Methods

This study aims to establish a developmental and growth profile for children with controlled CH in Egypt. The research also addresses gender-based differences in the development of children with CH. To achieve this, a case-control study was conducted to compare key parameters, including height, weight, receptive language, and expressive language, between children with CH and a group of age-matched healthy peers.

### Patients

A prospective case-control and follow-up study was carried out between February 2021 and February 2023 at the outpatient endocrinology pediatrics clinic of the 6th of October Health Insurance Hospital, Giza governorate, and the outpatient phoniatrics clinics at the Special Needs Center for Children at the Faculty of Post-graduate Childhood Studies, Ain Shams University. The study included 80 children aged 1.5 to 3 years, divided into two groups. Group A consisted of 40 children diagnosed with CH, and Group B consisted of 40 healthy peers. Both groups were matched for age and socio-economic status.

### Inclusion criteria for group A

Children in Group A were aged 1.5 to 3 years, of both genders, and had an early diagnosis of CH through neonatal screening. All children with CH attended the Pediatrics Endocrinology Committee at the 6th of October Health Insurance Hospital once a month for dose adjustment and clinical follow-up. These children were enrolled in the study between the ages of 1.5 to 3 years and underwent growth and developmental assessments three times: the initial assessment was conducted initially at time of first attendance, the first follow up was conducted after one year of initial assessment and the second follow-up occurred after two years of the initial assessment.

### Inclusion criteria for group B

Children in Group B were recruited from nurseries in the Giza Governorate. They had no concerns regarding growth and development and had normal thyroid function results as recorded in their Primary Health Care Cards. Both groups were subjected to follow-up assessments twice (in the same pattern as group A).

### Exclusion criteria

The exclusion criteria included prematurity, the presence of any comorbid conditions, the use of any current medical treatment for other health issues, and non-compliant patients with regard to treatment.

An informed assent for participation was obtained from the parents of all participating children. The researchers ensured the confidentiality of the data collected throughout the study. The study protocol adhered to human research ethics guidelines and was submitted for review to the Ethical Committee of the Faculty of Post-graduate Childhood Studies, Ain Shams University, where it received approval under ethical research committee approval number (FPGCS-ASUREC/RHDIRB2020110401/MSDFC-5/11/2021-S 17).

Sample size determination: The sample size was calculated to detect a moderate difference in certain growth and developmental parameters between children with congenital hypothyroidism and healthy controls. Assuming a medium effect size (Cohen’s d = 0.5), a confidence level of 95% (α = 0.05), and a statistical power of 80% (β = 0.2), the estimated required sample size was 63 children per group. To accommodate possible dropouts or incomplete data, this number was increased to 70 participants per group. However, due to practical constraints during recruitment, the final sample included 40 children with congenital hypothyroidism and 40 matched healthy controls. This sample size was considered adequate for preliminary analysis but may have limited the detection of smaller effect sizes.

### Setting of the study

The criteria for diagnosis of CH: CH was diagnosed at birth by newborn screening (NBS). The diagnosis of the positive cases for CH was further confirmed by simultaneous TSH and free T4 measurements. Both groups were subjected to 2 follow-up phases (the 1st is after 1 year, and the 2nd is after 2 years). The first follow-up was 1 year following the adjustment of the therapeutic dose of the thyroid hormones and confirming the adherence to treatment. At the same time, the second follow-up was 1 year following the first one.

#### Phase I

This phase involved the initial assessment protocol, which included detailed history taking through a structured interview questionnaire. The questionnaire was completed by the parents (or one of them) and covered the following items:

i Demographic data
  Demographic data included age, sex, and birth order, while sociodemographic data encompassed parental education, occupation, and consanguinity.
ii Risk factors
  Risk factors were categorized into several areas: prenatal care, which provided information on the care received during pregnancy; neonatal events, including mode of delivery, birth weight, gestational age at birth, and NICU admission; postnatal factors, such as type of feeding after birth, any birth injuries, jaundice, history of childhood illnesses, and hospital admissions; and family history, which focused on the presence of similar conditions in the family.
iii Complete clinical examination
  A thorough general examination was conducted, including the assessment of vital signs, clinical signs of CH, neurological examination (motor power, tone, and reflexes), abdominal examination, and cardiac examination.
iv Anthropometric Measurements
  Anthropometric measurements, including weight (kg), height (cm), and Body Mass Index (BMI = weight (kg)/height (m²)), were recorded and plotted on the growth chart using the Centers for Disease Control and Prevention (CDC) growth charts. These charts (released in 2000) are considered the standard for assessing growth patterns in children and adolescents, evaluating size and development based on age and sex [27].
v Developmental assessment
  - Language Assessment
    The Preschool Language Scale-4 (PLS-4) Arabic Edition was used to assess both receptive and expressive language abilities. The total language age was also determined through this assessment [28].
    Modified Preschool Language Scale-4 (Arabic Edition) (PLS-4) [28]: It has two standardized subscales and two supplemental measures. The two standardized subscales are the auditory comprehension subscale (the auditory comprehension subtest is composed of 62 items that are distributed in different age groups) and the expressive communication subscale (the expressive communication subtest is composed of 71 items that are also distributed in different age groups). The two supplemental measures are the articulation screener and caregiver questionnaire. The test proved to be valid as confirmed by internal consistency (internal consistency ranged from 0.991 to 0.998) and reliable (Crombach's alpha ranged between 0.60-0.92) to be applied on Egyptian children who are typically developing and those with delayed language development between the age of 2 months and 7 years and 5 months. Scoring: for each child, the basic and the ceiling level are determined, and the raw score is estimated by subtracting the ceiling score from the basal score. The raw score is converted into their equivalent language age in the conversion tables.  
  - Behavioral Assessment
    A behavioral assessment was conducted using the Portage Program Questionnaire, designed to evaluate developmental and behavioral aspects [29].
  - Cognitive Assessment
    Cognitive function was evaluated using the Stanford-Binet Intelligence Scales, Fifth Edition (Arabic Version), for children aged 2 to 5 years. This scale measures non-verbal, verbal, and full-scale intelligence [30]. For younger children (under 2 years), the Vineland Social Maturity Scale (VSM) was employed, which assesses adaptive behaviors across four subdomains: communication, social skills, daily living, and motor skills, and yields a composite adaptive behavioral score [31].
vi Laboratory Assessment
  Thyroid function was assessed by measuring Thyroid Stimulating Hormone (TSH) and free T4 (FT4) levels at baseline and after one year of treatment. Free hormone concentrations are considered more reflective of metabolic status than total hormone concentrations since they are unaffected by binding protein concentration or affinity. TSH normal values range from 0.5 to 5.0 mIU/L, while FT4 normal values range from 0.7 to 1.9 ng/dL. Free T3 (FT3) assays are typically unreliable and are not routinely used for thyroid function assessment [32].
vii Drug History
  Levothyroxine therapy was initiated immediately upon diagnosis, with a daily oral dosage of 10 to 15 mcg/kg. The dosage aimed to raise the serum T4 level to the upper half of the normal range for the child's age within 2 weeks and reduce TSH levels within 4 weeks. The dose was adjusted accordingly to maintain T4 and TSH levels within the normal range for the child's age [33].

#### Phase II of the study

Phase II involved re-applying the anthropometric and developmental assessments one year after the initial evaluation.

#### Phase III of the study

Phase III entailed re-assessing both the anthropometric and developmental parameters two years after the initial assessment.

#### Data management and analysis

The collected data were carefully reviewed, coded, and tabulated before being entered into a computer using the Statistical Package for the Social Sciences. Data were presented and analyzed based on the specific type of data for each parameter.

#### Descriptive statistics

The descriptive statistics included the mean, median, standard deviation (± SD), minimum and maximum values (range) for numerical data, and 95% confidence intervals (CI). For categorical data, frequency and percentage were calculated.

#### Analytical statistics

The normality of distribution parameters was initially evaluated using the One-Sample Kolmogorov-Smirnov test. For data with a normal distribution, the Independent-Samples T-Test was used to assess the statistical significance of differences between the means of two study groups, while the Paired-Samples T-Test evaluated differences in a single quantitative variable measured at two time points within the same group. The One-Way ANOVA was employed to determine statistical significance among the means of more than two groups. Pearson’s correlation coefficient (r) was used to measure the strength and direction of linear relationships between two quantitative variables. For nonparametric distributions, the Mann-Whitney U Test and Kruskal-Wallis Test were applied. Spearman’s rank correlation coefficient (ρ) assessed the strength and direction of associations between two quantitative variables in nonparametric data. For qualitative data, the Chi-Square Test examined relationships between variables, while Fisher’s Exact Test was applied when expected frequencies in contingency table cells were less than five. Statistical significance was determined as follows: *P* > 0.05 was considered not significant (NS), *P* ≤ 0.05 was significant (S), and *P* ≤ 0.01 was highly significant (HS).

## Results

### Descriptive statistics

The current study included a sample of 80 Egyptian children, comprising 63 males (78.8%) and 17 females (21.3%), aged between 1.5 and 3 years, with a mean age of 2 years and 4 months (± 0.5). The children were divided into two groups: Group A, consisting of 40 children with CH, and Group B, consisting of 40 healthy control children. Both groups were matched for mean age, gender distribution, and socio-economic status, as determined using the socio-economic and cultural standard scale developed by Saffan & Khattab [34].

Regarding maternal characteristics, there were notable differences between the two groups. While the ages of the mothers in both groups were similar, several unfavourable perinatal conditions were more prevalent in Group A. These included a lack of antenatal care, reported in 75% of mothers in Group A compared to only 5% in Group B, consanguineous marriages (42% in Group A vs. 12.5% in Group B), and a lower maternal education level (40% of mothers in Group A were illiterate, compared to 7.5% in Group B).

### Comparative statistics

#### 1st Follow-up

The data revealed no significant difference between children in Group A and Group B regarding weight, height, receptive and expressive language ages, and behavioral profiles after the first follow-up. These findings are presented in Table 1.

**Table 1:** Compared growth and development parameters of children in group A and group B after the first and second follow-up

Growth and developmental parameters	Groups	N	Mean	SD	Median	Range		T	P Value	Sig.
						Min.	Max.			
Height	Group B 1^st^ FU.	40	88.4	5.1	88.5	74.0	98.0	0.35	0.724	NS^*^
	Group A 1^st^FU.	40	88.0	5.0	87.0	79.0	100.0			
Weight	Group B 1^st^ FU.	40	12.4	1.6	12.5	10.0	16.0	1.58	0.118	NS^*^
	Group A 1^st^FU.	40	13.1	1.9	13.0	10.0	18.0			
Receptive language age	Group B 1^st^ FU.	40	2.2	0.6	2.3	1.1	3.4	1.85	0.069	NS^*^
	Group A 1^st^FU.	40	2.0	0.5	2.0	1.1	3.1			
expressive language age	Group B 1^st^ FU.	40	2.1	0.6	2.2	1.2	3.3	1.81	0.074	NS^*^
	Group A 1^st^FU.	40	1.9	0.5	1.9	1.1	3.2			
Height	Group B 2^nd^ FU.	40	97.5	2.5	98.0	93.0	103.0	4.82	<0.001	HS^**^
	Group A 2^nd ^FU.	40	93.7	4.3	93.5	84.0	106.0			
Weight	Group B 2^nd^ FU.	40	17.0	1.1	17.2	15.2	19.2	3.38	0.001	HS^**^
	Group A 2^nd^FU.	40	15.7	2.3	15.0	13.0	22.0			
Receptive language age	Group B 2^nd^ FU.	40	3.6	0.5	3.7	2.8	4.7	5.01	<0.001	HS^**^
	Group A 2^nd ^FU.	40	3.1	0.5	3.0	2.1	4.5			
expressive language age	Group B 2^nd^ FU.	40	3.3	0.6	3.2	2.1	4.6	2.87	0.005	HS^**^
	Group A 2^nd ^FU.	40	3.0	0.6	2.9	2.1	4.3			

The mean value and the standard deviation of height, weight, receptive language age, and expressive language age were compared after the first and second year of life. The table showed that there is a significant difference between the mean value of different growth and developmental parameters after the 2nd follow-up. Independent-Samples T test *P*>0.05=NS, *P*<0.05=S,* P*<0.01=HS was used

*SD* Standard deviation, *FU *Follow-Up, NS* Non-significant, HS** Highly significant

#### **2nd Follow-up**

After two years of the initial assessment, Group A exhibited significantly lower scores in receptive and expressive language, as well as in height and weight, compared to their healthy peers in Group B. The *p*-value was < 0.001. These results are shown in Table 1.

Additionally, the growth chart after the second year of assessment revealed that approximately 60% of children in Group A were stunted, and 7.5% were overweight. This data is illustrated in Table 2.

**Table 2:** The frequency of different growth patterns in height and weight in group B and group A children

Variable	Description of gain in height and weight	Group B(n=40)	1^st^ FU group A(n=40)	2^nd^ FU group A(n=40)
Growth chart for height	Normal height(-1SD to+2SD)	19 (47.5%)	22 (55.0%)	12(30.0%)
	Mild stunted(-2SD to -1SD)	19 (47.5%)	11 (27.5%)	17(42.5%)
	Moderate stunted (-3SD to -2SD)	1 (2.5%)	6 (15.0%)	8 (20.0%)
	Severely stunted(<-3SD)	1 (2.5%)	1 (2.5%)	3 (7.5%)
Growth chart for weight	Normal weight (25%-75%)	9 (22.5%)	19 (47.5%)	21(52.5%)
	Mild underweight (<25%)	15 (37.5%)	12 (30.0%)	12(30.0%)
	Moderate underweight (<10%)	14 (35.0%)	6 (15.0%)	4 (10.0%)
	Severely underweight (<3%)	2 (5.0%)	0	0
	Over weight (>75%)	0	3 (7.5%)	3 (7.5%)

The table showed data regarding height and weight as plotted on growth charts to assess both normal and abnormal growth patterns among children in group A and children in group B. Fisher's Exact Chi-Square Test was used

The mean percentage increase across all domains was calculated for both groups after the second follow-up. The percentage increase was significantly higher in Group B, particularly in the height and weight domains, with a *p*-value of < 0.001. The results are shown in Table 3.

**Table 3:** The mean of percentage increase in growth and development parameters for children in group B and group A after the second follow-up

Growth and developmental domains	Groups	N	Mean%	SD	Median	Range		T	*P* Value	Sig.
						Min.	Max.			
Height	Group B	40	10.6	5.2	10.8	.0	27.7	4.28	<0.001	HS^**^
	Group A	40	6.7	2.8	6.9	.0	17.7			
Weight	Group B	40	38.7	14.1	37.0	10.0	72.0	3.92	<0.001	HS^**^
	Group A	40	22.3	22.5	16.7	18.8	80.0			
Expressive language age	Group B	40	60.4	27.5	55.3	11.1	116.7	0.07	0.944	NS
	Group A	40	59.9	35.4	54.2	5.0	170.3			
Receptive language age	Group B	40	70.5	40.0	65.2	35.2	224.3	1.76	0.078	NS
	Group A	40	60.7	34.3	52.9	3.7	172.7			

The table compared the percent increase in the mean value of height, weight, receptive language age, and expressive language age in group A and group B during the second follow-up in group A children and group B. There was a significantly higher mean in group B than in group A children in terms of growth parameters (weight and height). However, this significance was absent when comparing the mean percent increase in receptive and expressive language ages. T=Independent-Samples T test, Z= Mann Whitney Test, *P*>0.05=NS, *P*<0.05=S, *P*<0.01=HS.

*NS *Non-significant, HS** Highly significant

#### Gender differences

Data from the current study revealed that male children with CH presented at the pediatric endocrinology clinic in greater numbers than females. The demographic characteristics of males and females in Group A were similar to those of the overall study population. However, developmental profiles between male and female children differed at the first follow-up; female children with CH exhibited lower receptive language age, expressive language age, and total language age compared to their healthy peers. These findings are shown in Table 4. In contrast, male children with CH showed developmental outcomes that closely resembled those of their healthy peers. These results are presented in Table 4.

**Table 4:** Compared growth and development parameters of female and male children in group A and group B after the 1st and 2nd follow-up

Variables		Groups	N	Mean	SD	Median	Range		T	P Value	Significance
							Min.	Max.			
Receptive language age	Female	Group B 1^st^ FU	10	2.6	0.5	2.6	1.8	3.4	2.5	0.025	S^*^
		Group A 1^st^ FU	7	2	0.5	2	1.1	2.8			
	Male	Group B 1^st^ FU	30	2.1	0.6	2.2	1.1	2.9	0.7	0.484	NS
		Group A 1^st^ FU	33	2.0	0.5	2.0	1.1	3.1			
Expressive language age	Female	Group B 1^st^ FU	10	2.5	0.6	2.5	1.7	3.3	2.7	0.015	S^*^
		Group A 1^st^ FU	7	1.7	0.6	1.6	1.1	2.7			
	Male	Group B 1^st^ FU	30	2.0	0.5	2.1	1.2	2.8	0.46	0.645	NS
		Group A 1^st^ FU	33	2.0	0.5	1.9	1.1	3.2			
Total language age	Female	Group B 1^st^ FU	10	2.5	0.6	2.5	1.7	3.3	2.6	0.018	S^*^
		Group A 1^st^ FU	7	1.8	0.6	1.7	1.1	2.7			
	Male	Group B 1^st^ FU	30	2.1	0.5	2.1	1.1	2.9	0.79	0.431	NS
		Group A 1^st^ FU	33	2.0	0.5	1.8	1.1	3.1			
Height	Female	Group B 2^nd^ FU	10	98.9	2.6	99	95	103	4.2	0.001	HS^**^
		Group A 2^nd^ FU	7	93.3	2.8	93	90	98			
	Male	Group B 2^nd^ FU	30	97.1	2.4	98.0	93.0	100	3.4	0.001	HS^**^
		Group A 2^nd^ FU	33	93.8	4.6	94.0	84.0	106			
Weight	Female	Group B 2^nd^ FU	10	17.6	1.1	17.4	16	19.2	2.9	0.012	S^*^
		Group A 2^nd^ FU	7	15.9	1.3	16	14	18			
	Male	Group B 2^nd^ FU	30	16.9	1.0	17.2	15.2	19.0	2.5	0.016	S^*^
		Group A 2^nd^ FU	33	15.7	2.5	15.0	13.0	22.0			
Receptive language	Female	Group B 2^nd^ FU	10	3.9	0.6	4	3	4.7	3.6	0.003	HS^**^
		Group A 2^nd^ FU	7	3	0.3	3	2.8	3.7			
	Male	Group B 2^nd^ FU	30	3.5	0.4	3.6	2.8	4.3	3.6	0.001	HS^**^
		Group A 2^nd^ FU	33	3.1	0.5	3.0	2.1	4.5			
Expressive language	Female	Group B 2^nd^ FU	10	3.5	0.7	3.8	2.1	4.6	2.1	0.051	NS
		Group A 2nd FU	7	2.9	0.5	2.8	2.1	3.7			
	Male	Group B 2^nd^ FU	30	3.2	0.5	3.2	2.1	4.0	1.9	0.058	NS
		Group A 2^nd^ FU	33	3.0	0.6	3.0	2.1	4.3			

The table compared: 1- Female children in groups A and B concerning growth and development parameters in the 1^st^   and 2^nd^ follow-up. The table showed that there is a significant difference between the mean value of different developmental parameters (receptive language age, expressive language age, and total language age) after the 1^st^ follow-up. This difference was maintained except for expressive language skills. Independent-Samples T test *P*>0.05=NS, *P*<0.05=S, *P*<0.01=HS

2- Male children in group A and B concerning growth and development parameters in the 1^st^ and 2^nd^ follow-up. The table compared male children in group A and B concerning growth and development parameters in the 1^st^   and 2^nd^ follow-up. The table showed that there is no significant difference between the mean value of different developmental parameters (receptive language age, expressive language age, and total language age) after the 1^st^ follow-up. However, after the 2^nd^ follow-up, the significance of the difference between the mean values of different growth and developmental parameters appeared, except for expressive language skills. Independent-Samples T test *P*>0.05=NS, *P*<0.05=S, *P*<0.01=HS

*SD *standard deviation, *Min *minimum, *Max *maximum, *FU* Follow-Up, *NS *Non-significant, S^*^ Significant,HS^**^Highly significant

By the second follow-up, both female and male children with CH demonstrated a similar developmental profile. Both groups showed significant differences when compared to their healthy peers across all assessed domains, including receptive language age, height, and weight. The p-values for females were 0.001, 0.012, and 0.003, and for males, the p-values were 0.001, 0.016, and 0.001, respectively.

The mean percentage increase in height, weight, receptive language age, and expressive language age was notably different between genders. Data shown in Table 5 highlighted the developmental progress for each gender. Female children with CH showed a significant increase in expressive language abilities after the second follow-up compared to their healthy peers, suggesting a notable developmental spurt. Conversely, male children with CH were significantly lower in height and weight, even after controlling for CH, in comparison to their healthy peers. 

**Table 5:** The mean of percentage increase in growth and development parameters for female and male children in group B and group A

Variables		Groups	N	Mean%	SD	Median	Range		t	P Value	Sig.
							Min.	Max.			
Height	Female	Group B	10	9.4	4	8.7	5.3	17.9	1.73	0.105	NS
		Group A	7	6.6	2	6.9	3.2	9.4			
	Male	Group B	30	11.0	5.5	11.1	.0	27.7	3.97	<0.001	HS^*^^*^
		Group A	33	6.7	3.0	6.9	.0	17.7			
Weight	Female	Group B	10	36.4	17.1	35.2	10	60	0.01	0.994	NS
		Group A	7	36.5	17.7	33.3	15.4	63.6			
	Male	Group B	30	39.5	13.1	37.6	14.3	72.0	4.30	<0.001	HS^**^
		Group A	33	19.2	22.5	16.7	-18.8	80.0			
Receptive language age	Female	Group B	10	51.4	11.2	52.9	35.3	66.7	0.85	0.408	NS
		Group A	7	63.7	44.1	55	32.1	161.3			
	Male	Group B	30	76.8	44.1	71.1	35.2	224.3	1.73	0.089	NS
		Group A	33	60.0	32.7	52.9	3.7	172.7			
Expressive language age	Female	Group B	10	43.7	15	43.8	24.1	70.6	2.14	0.049	S^*^
		Group A	7	76.6	45.6	64.7	36.4	170.3			
	Male	Group B	30	65.9	28.6	61.3	11.1	116.7	1.24	0.221	NS
		Group A	33	56.3	32.5	52.4	5.0	170.3			

The table compared: 1- The percent increase in the mean value of height, weight, receptive language age, and expressive language age among female children in group A and group B during the second follow-up. There was a significantly higher mean in group B than in group A in expressive language ages. T=Independent-Samples T test, Z= Mann Whitney Test, *P*>0.05=NS, *P*<0.05=S, *P*<0.01=HS

2- The percent increase in the mean value of height, weight, receptive language age, and expressive language age among male children in group A and group B during the second follow-up. There was a significantly higher mean in group B than in group A in terms of height and weight. T= Independent-Samples T test, *P*>0.05=NS, *P*<0.05=S, *P*<0.01=HS

S^*^ significant increase

### Correlative statistics

The correlative analysis revealed that the mean value of percentage increase in height, weight, and expressive and receptive language age from the first to the second follow-up was not significantly correlated with variables such as TSH at birth or Free T4 levels. However, a noteworthy inverse correlation was found between the children’s age in Group A and the percentage change in receptive language age scores. Specifically, the older the children in Group A, the smaller the increase in their receptive language score. Data are shown in Table 6. 

**Table 6:** Correlation between the mean of percent increase in height, weight, receptive language age, and expressive language age in group A and some independent variables

Independent variables	The correlation	The mean of % increase of height	The mean of % increase of weight	The mean of % increase expressive language age	The mean of % increase in receptive language age
TSH* at birth	r	0.29	0.14	-0.05	0.16
	P value	0.065	0.396	0.769	0.323
	Sig.	NS	NS	NS	NS
Free T4	r	0.05	-0.05	-0.10	-0.31
	P value	0.775	0.736	0.533	0.055
	Sig.	NS	NS	NS	NS
Age	r	-0.13	0.09	-0.13	-0.49
	P value	0.440	0.561	0.416	0.001
	Sig.	NS	NS	NS	HS**

The table correlated between the mean values of percent increase in height, weight, receptive language age, and expressive language age in group A and some independent variables. Pearson correlation=r, Spearman's Correlations = rho, *P*>0.05=NS, *P*<0.05=S, *P*<0.01=HS

*TSH *Thyroid stimulating hormone

## Discussion

The neonatal screening program for inborn errors of metabolism, including CH, has made significant strides in providing early detection and intervention, covering more than 90% of Egyptian children with CH. Despite this, such measures do not guarantee that these children will achieve developmental milestones comparable to their typically developing peers [4, 8, 35].

In the current study, 78.8% of the cases were male, a finding consistent with Zimmermann [36], who noted a slightly higher incidence of CH in males. This discrepancy may be attributed to sex-linked genetic factors or differences in screening sensitivity between genders. However, Nili et al. [37] reported a nearly equal prevalence between genders. A study in Upper Egypt by Habib et al. [27] found that 52.5% of the CH cases were female. A meta-analysis by Rezaeian et al. [38] suggested that gender remains an unknown risk factor for CH among children. The analysis of gender differences in CH suggests that the type of CH may influence gender prevalence. Permanent CH is more commonly seen in females, while the transient form tends to be more prevalent in males. Other studies have explained these inconsistencies by factors such as sample size, chance, study design, population variability, and different statistical methods [26].

The current study found that approximately 50% of children in group A were from consanguineous marriages, a result that aligns with findings by Hashimpour et al. [39]. Stoupa et al. [40] also highlighted the potential genetic component of CH, noting that consanguinity increases the likelihood of inheriting recessive genetic mutations. The data further indicated that antenatal care was inadequate in group A. Previous research has emphasized the importance of antenatal care in preventing and managing congenital conditions. Providing early intervention and educating expectant mothers about the potential risks can help reduce the likelihood of developmental issues later in life [41].

### Language development

A significant finding of this study is the limited language acquisition abilities observed among children with CH (despite average cognitive abilities). This result is consistent with various studies conducted both in Egypt [27, 42] and internationally, such as Menotti et al. [43]. This suggests that CH may have a persistent impact on language development, despite regular treatment. It highlights the need for ongoing developmental support, as well as potentially more intensive and targeted interventions to address specific language challenges in these children. In the current study, the second follow-up revealed significant differences in receptive and expressive language scores between children with CH and their healthy peers. Similarly, Romero et al. [44] found that while early language skills were comparable, children with CH experienced a slower rate of development in these areas over time. The authors recommended early language intervention for children with CH to address these delays.

In addition to the critical role of thyroid hormones in the development of the brain cortex, studies by Inglesby et al. [45], Fang et al. [46], and Hussien et al. [47] have shown that early hormonal deficiencies can lead to impaired structures and functions of the inner ear. Poonual et al. [48] further reported that children with CH are 2.5 times more likely to develop sensorineural hearing loss (SNHL) compared to children without hormonal deficiencies. Braga et al. [49] also found that children with uncontrolled CH are at an increased risk of functional deficits, even without deteriorating hearing sensitivity. These findings may help explain the decline in receptive language abilities observed in children with CH in the current study. However, van Trotsenburg et al. [50] disagreed with the current findings, suggesting that early and adequate treatment for CH can lead to language development outcomes similar to those of healthy children. The discrepancy may be attributed to the more detailed and objective assessment of language skills employed in the present work, which could capture subtler developmental differences.

An earlier study by Mohamed et al. [42] in El-Menia Governorate concluded that even when CH is controlled, language and IQ scores remain lower than those of matched healthy peers. Unfortunately, this study did not include an assessment of language age, which may have provided more comprehensive insights into language development.

On the other hand, Gejão et al. [26] found that children with CH, aged between 2 and 36 months, showed significant deficits, especially in expressive language and cognitive abilities, as measured by the Early Language Milestone Scale (ELM) and the Portage Operations Inventory. The multifactorial nature of language deficits in children with CH has also been discussed by Razón-Hernández et al. [24]. The author highlighted that memory deficits and the increased complexity of language tests contribute to these deficits. The timing of hormonal therapy replacement was identified as a pivotal factor in language development, with a strong correlation found between fine motor deficits and phonological deficits [51, 52]. This suggests that a comprehensive approach to treatment that includes both hormonal therapy and developmental interventions is necessary to optimize outcomes for children with CH.

### Height and weight development

The current study observed an increase in height and weight following the second follow-up, which is consistent with other findings [53]. However, it also revealed a concerning trend in the growth chart of children with CH, showing a decline in the percentage of children with normal height, from 55% in the first follow-up to 30% in the second. Additionally, around 8% of children with CH exhibited severe stunted stature, compared to only 2.5% of their typically developing peers. These findings align with other studies that, while initial growth metrics in children with CH may be comparable to those of typically developing peers, growth in height and weight tends to slow over time [54, 55]. This could reflect the long-term effects of CH on growth, particularly linear growth and bone development, despite initial treatment.

Regarding weight, the growth chart data in the present study indicated a tendency for children with CH to become overweight. By the second follow-up, 7.5% of children with CH were overweight, compared to none in the control group. This finding supports the observations made by Habib et al. [27], suggesting that children with CH may be at risk for weight-related issues as they age. The current study also found that growth and developmental deficits became more apparent in the second follow-up among children with CH. This is in line with a study conducted by Abdelmoktader [11] in Ismalia, where children with controlled CH showed only 20% improvement in weight-for-age and 26.7% improvement in height-for-age.

Contrary to these findings, Habib et al. [27] reported that growth and developmental deficits were more common in their younger participants. They suggested that the severity of CH is linked to cognitive and motor outcomes, highlighting the importance of early diagnosis and intervention. However, Heidari et al. [56] offered a different perspective, stating that the early administration of T4 has a strong initial effect on growth, particularly in terms of height. This underscores the need for timely and effective treatment to mitigate the long-term impacts of CH on growth and development.

### Cognitive abilities and CH

The current study found that the cognitive abilities of children with CH closely approximated those of their healthy peers. This is in agreement with the work of Grosse & Van [57], who found that at age 2, children with CH exhibited cognitive development comparable to that of typically developing children. However, by later stages, approximately 28% of children with CH showed a leftward shift of one standard deviation in their cognitive abilities. This finding is consistent with those of Bongers-Schokking et al. [58] and Nili et al. [37], who reported similar cognitive outcomes, with IQ scores among children with CH being nearly identical to those of their age-matched healthy peers (104.7 ± 16.2 vs. 105.0 ± 15.8), regardless of the type of CH (permanent or transient). In contrast, Arad et al. [59] observed a somewhat different pattern. In their study of 6-year-old children with CH, they found that 73% of the children had average or above-average IQ scores, while the remaining 27% exhibited cognitive deficits, with 14% showing borderline IQ and 12% demonstrating mild mental retardation (MR). Arad et al. concluded that cognitive deficits in children with CH were associated with low thyroid hormone levels during the perinatal and early neonatal periods. These conflicting findings may be attributed to the differences in the mean age of the samples studied, with the older children in Arad et al.‘s study potentially showing more pronounced cognitive deficits due to prolonged hormonal deficiency during critical developmental periods.

In summary, while the cognitive abilities of children with CH are generally comparable to their peers, the findings highlight that the long-term effects of CH may become more evident as children age, with those who experienced significant hormonal deficiency in early life potentially at greater risk for cognitive delays. The factors influencing IQ scores among children with CH are multifaceted. A consistent finding across studies is the importance of the age at which treatment begins and the dosage of hormonal replacement therapy. Nili et al. [37] further suggested that children with controlled CH, whose mothers had low educational levels, received inadequate antenatal care and experienced hazardous deliveries, and tended to show lower cognitive abilities. They argued that when socio-economic factors are controlled for, the cognitive differences between children with CH and healthy children become less significant.

Additionally, Dimitropoulos et al. [60] highlighted that, over the long term, intellectual abilities in adolescents with CH are closely linked to the underlying cause of CH and socio-economic status. The better outcomes observed in the current study may be attributed to the younger age of the children in the sample and the stimulating social and environmental context in Egypt. Habib et al. [27] explained that higher developmental scores were found in children from urban areas, where the availability of nurseries and access to a more stimulating environment facilitated better skill acquisition.

Regarding gender differences, the current study found that female children with CH showed early (after the first follow-up) significantly lower receptive and expressive language abilities compared to their healthy peers during the first follow-up. In contrast, male children with CH exhibited more significant delays in growth (height and weight) compared to their healthy counterparts. This gender-based divergence in developmental profiles aligns with the findings of Habib et al. [27], who also reported gender differences in the impact of CH on growth and language development. These findings emphasize the importance of early detection, hormonal treatment, and a supportive environment for children with CH, as well as the need to consider gender and socio-economic factors when evaluating developmental outcomes.

In a similar vein, Razavi et al. [61] identified that problem-solving abilities were the most frequently affected domain among girls with CH, while physical development issues were more commonly observed among boys. The findings of the current study may be explained by variations in the diagnosis among the affected children. Some children in the sample may have been diagnosed with TCH, while others had the permanent form. TCH tends to have a better prognosis, but this distinction could not be proved in the current study due to the retrospective nature of the diagnosis and the young age of the children (mean age: 2 years and 4 months).

The variability in the presentation of growth and developmental deficits among children with CH was comprehensively discussed by Razón-Hernández et al. [24], who noted that over 85% of individuals treated for CH will experience neuropsychological sequelae throughout their lives. The aetiology of these alterations remains unknown, but factors contributing to neuropsychological changes are categorized into intrinsic and extrinsic factors. Extrinsic factors include social influences, such as socio-economic status, family environment, caregiver characteristics, and treatment-related factors like timing of treatment initiation, initial dosage, monitoring, dose adjustments, and treatment adherence. Intrinsic factors involve the severity of CH, anatomical and physiological changes in specific brain regions, and thyroid physiological alterations in the brain, such as mild thyroid hyposensitivity, which impairs thyroid hormone mechanisms within the brain. The future holds promise for advancing the understanding of CH’s pathophysiology at both the genetic and biochemical levels, which could pave the way for new therapeutic strategies.

Regarding the correlative statistics from the current study, an inverse correlation was found between age and the percentage of change in receptive language. This finding aligns with the work of Habib et al. [27], who suggested that T4 and TSH levels did not significantly impact growth and development in children with CH, emphasizing that other factors might play a more significant role in shaping developmental outcomes.

## Conclusion

Congenital hypothyroidism (CH) remains a significant paediatric endocrine disorder with profound implications for both intellectual and physical development. In this study, despite early initiation of therapy, close monitoring of dose adjustments, and adequate treatment compliance, the second follow-up showed significant deficits in height, weight, and receptive language age among children with CH. Girls, in particular, demonstrated earlier and more frequent deficits across developmental domains. This highlights the need for ongoing, tailored interventions for this population.

### Recommendations

A key strength of this study lies in the collaborative approach between clinical care and objective assessment of children with CH by both phoniatricians and pediatricians. This integrated method should be expanded to other disciplines across Egypt to ensure comprehensive management of CH. Children with CH require systematic follow-up of developmental milestones using objective and standardized tools to detect early signs of developmental delay, its type, and severity. Based on these comprehensive assessments, a multidisciplinary team should provide appropriate and individualized rehabilitation programs.

Future research should focus on understanding the reasons behind the variability in growth and developmental alterations among children with CH. A specific developmental profile should be established for different Egyptian governorates, including data on CH types, severity, patient compliance, and growth and development charts. Such profiles would enable authorities to offer more targeted support for assessment and rehabilitation services. Future work should also encourage long-term follow-up studies to further elucidate the long-term impact of CH treatment on cognitive and physical development over time.

### Limitations of the study

This study was limited to children in Giza governorate. Further research should include other governorates across Egypt, with a larger sample size and inclusion of different age groups (e.g., infants, preschool children, and school-aged children) to enhance the generalizability of the findings. The socioeconomic difference between groups should be addressed in future studies. In addition to this, other developmental areas are strongly recommended for assessment which included academic performance in older children.

## Data Availability

The data supporting the conclusions of this article are available on reasonable request from the corresponding authors on contacting: email: hassnaaphoniatrics@gmail.com.

## Abbreviations

CH: Congenital hypothyroidism
NICU: Neonatal intensive care admission
TCH: Transient hypothyroidism
T4: Thyroxin
TSH: Thyroid stimulating hormone
CDC: Centers for Disease Control and Prevention growth charts
PLS-4 (Arabic Edition): The Preschool Language Scale-4 Arabic edition
FT3: Free T3
SPSS: Statistical package for social science
ELM: Early Language Milestone Scale
IQ: Intelligent Quotient
